# Vascularized Versus Non-vascularized Bone Grafting for Scaphoid Non-union: A Meta-Analysis

**DOI:** 10.7759/cureus.77711

**Published:** 2025-01-20

**Authors:** Abhishek Guria, Kumar Vaibhav, Navin Kumar, Saumya Kullu

**Affiliations:** 1 Orthopaedics, Rajendra Institute of Medical Sciences, Ranchi, IND

**Keywords:** meta-analysis, non-vascularized bone grafting, scaphoid nonunion, scaphoid pseudoarthrosis, union rate, vascularized bone grafting

## Abstract

Non-union after scaphoid fracture is a common complication. Controversy exists regarding the efficacy of vascularized bone graft (VBG) versus non-vascularized bone graft (NVBG) for scaphoid non-union. In the current meta-analysis, a comprehensive search of PubMed and the Cochrane Library database was done from inception to June 2024. We included randomized controlled trials (RCTs) and prospective comparative studies that reported outcomes following scaphoid non-union comparing VBG versus NVBG. Two reviewers independently extracted data and assessed the risk of bias. Any discrepancy was resolved by consensus and discussion with a third reviewer. We included five RCTs and one prospective comparative study. The combined odds ratio is 1.57 (95% CI: 0.87-2.86), favoring a higher union rate in the experimental group. The overall pooled mean difference of -1.40 (95% CI: -2.03 to -0.77) indicated that the VBG has a significantly shorter time to union compared to the NVBG. In conclusion, our results showed that VBG may be associated with an improved union rate and a decrease in union time, though no significant functional benefits.

## Introduction and background

Scaphoid fractures form 50-80% of all carpal fractures [1,2]. Most of the scaphoid fractures heal, though a number of case series have identified a non-union rate of 10-15% [1]. Non-union mostly occurs due to the disruption of the blood supply of the scaphoid. Fracture site location, soft tissue interposition, and inadequate immobilization predispose to non-union [3]. Following non-union of the scaphoid, progressive degeneration occurs at the site of the fracture in the form of a cyst, bone loss, and development of apex, dorsal angulation, and humpback deformity [4]. This may lead to scaphoid non-union advanced collapse (SNAC) of the wrist and the formation of a proximal pole, which extends with the lunate. The resultant wrist architecture is known as dorsal intercalated segment instability (DISI) deformity [4,5]. Scaphoid non-union causes pain and decreased movement of the wrist, along with diminished grip strength [4]. The treatment aims at pain relief, increased wrist function, and prevention of secondary osteoarthritis [6].

The management of scaphoid non-union has been controversial. Internal fixation with bone grafting is the current recommended treatment [7,8]. Non-vascularized bone graft (NVBG) comprising cortical or cancellous bone, extracted from autograft or allograft, combined with fixation had significant improvement in union rate [9]. In the presence of avascular necrosis (AVN), NVBG achieved union in 47% of cases and 94% where there was no AVN [10]. These findings signified the importance of the vascular supply of the scaphoid bone. This belief that adequate blood supply would facilitate union vascularized bone grafting (VBG) was stemmed. VBGs were further classified into free or pedicled VBGs. Local VBG is harvested from a distal radius, either dorsally pedicled on the 1,2-intercompartmental supraretinacular artery (1,2-ICSRA) [11] or volarly pedicled on the volar carpal artery [12]. Recently, many surgeons have used free VBG from the medial femoral condyle or iliac crest [13]. No concrete evidence has been provided for treatment regarding which technique is more effective [10]. This meta-analysis aims to provide evidence-based suggestions in choosing graft type for the management of scaphoid non-union.

## Review

Methods

Literature Search Strategy

As the data extracted was from already published articles, ethical committee approval was not required for this study. The present review was registered in the International Prospective Register of Systematic Reviews (PROSPERO) (CRD42024565021) and carried out in view of the recent Preferred Reporting Items for Systematic Reviews and Meta-Analyses (PRISMA) guidelines. Electronic database searches were from PubMed and the Cochrane Library database. We employed the terms "scaphoid non-union", "scaphoid pseudoarthrosis", and "bone graft" to search for the studies. Two authors independently reviewed the studies published till June 2024, and in case of any discrepancy, a thorough discussion with the third investigator was done. All articles that met the inclusion criteria for the studies were included. Articles were excluded if they came under the exclusion criteria.

Inclusion Criteria

Studies were selected if they fulfilled the inclusion criteria as follows: (i) English literature, (ii) randomized and prospective comparative studies, (iii) patient cohort comprising scaphoid non-union or scaphoid pseudoarthrosis, (iv) full-length articles in which patients with scaphoid non-union treated with VBG and NVBG were compared, and (v) articles having outcome data, union rate, union time, and function outcome.

Exclusion Criteria

Articles were excluded on the basis of the following exclusion criteria: (i) non-English literature; (ii) review articles, conference papers, case reports, tutorials, surgical techniques, and retrospective studies; (iii) studies not comparing VBGs and NVBGs; and (iv) studies not having outcome data: union rate, time to union, and function outcome.

Data Extraction

Two investigators independently extracted the data from eligible studies using determined the selection criteria. Disagreements were resolved through a discussion with a third investigator. The data extracted from studies included authors, publication year, country, number of participants, patient population, intervention, follow-up time, and end results.

Assessment of Methodical Quality

Two authors independently assessed the methodological quality of each included article. Disagreements were dissolved through discussion and consensus. Randomized controlled studies were assessed according to the Cochrane Handbook for Systematic Reviews of Interventions. The risk of bias in a randomized controlled trial (RCT) was evaluated using the RevMan software (The Cochrane Collaboration, London, England, United Kingdom), which included key domains: random sequence generation, allocation concealment, blinding of participants and personnel, blinding of outcome assessment, incomplete outcome data, and selective reporting.

Statistical Analysis

We performed statistical analysis using IBM SPSS Statistics for Windows, Version 21.0 (Released 2012; IBM Corp., Armonk, New York, United States) [14]. Continuous variables were analyzed using weighted mean difference, whereas dichotomous variables were analyzed using relative risk. A p-value of less than 0.05 was statistically significant, and 95% confidence intervals were reported. Heterogeneity was tested by the Q statistic and the I2 statistic. The fixed-effect model was used when the test for heterogeneity was not significant (p>0.1; I2<50%), while p<0.1 or I2>50% was considered suggestive of statistical heterogeneity, and the random-effects model was used.

Results

Study Selection

**Figure 1:**
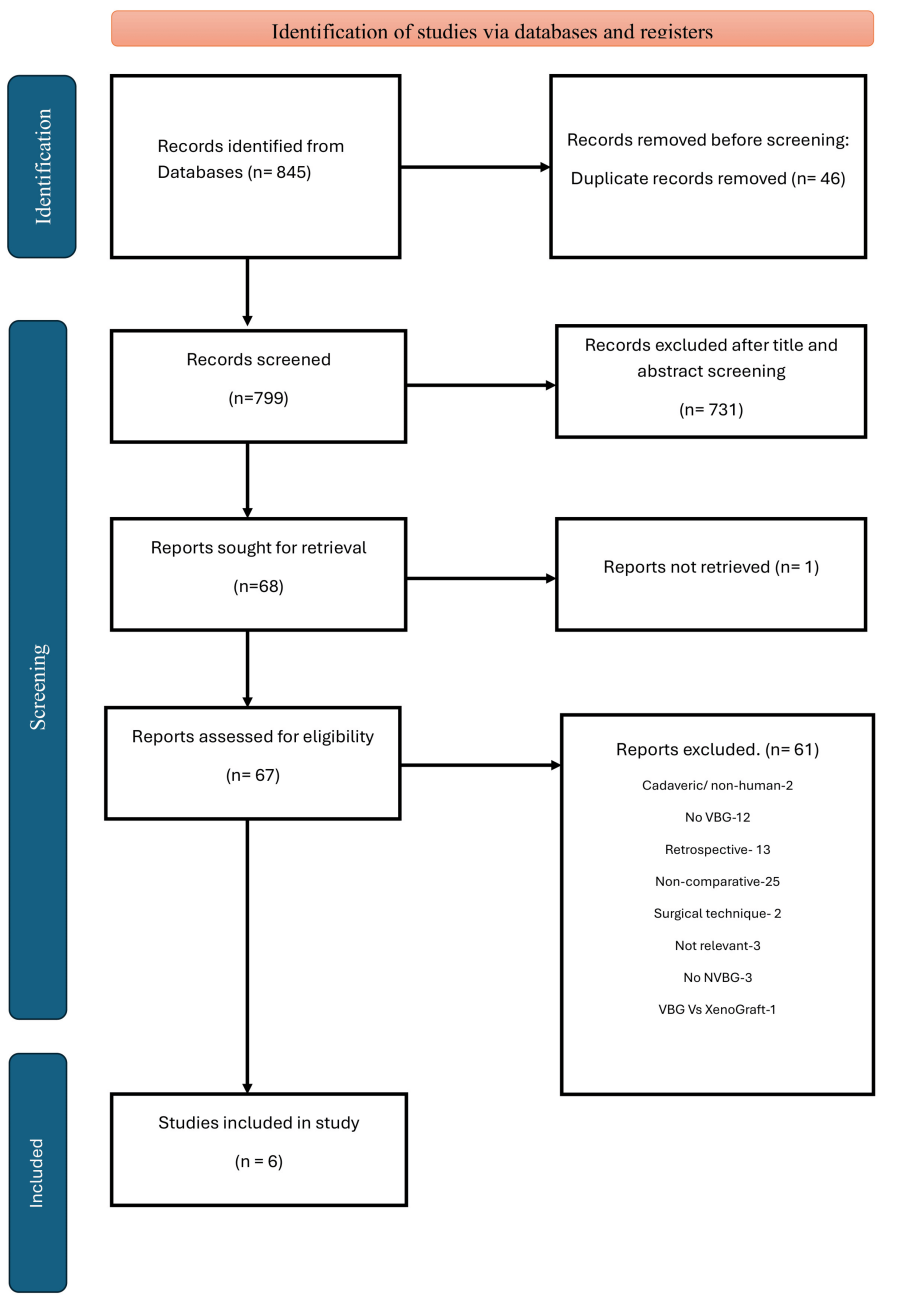
PRISMA flowchart of study selection PRISMA: Preferred Reporting Items for Systematic Reviews and Meta-Analyses; VBG: vascularized bone graft; NVBG: non-vascularized bone graft

Our search resulted in 764 articles from PubMed and 81 articles from the Cochrane Library, from which 46 duplicate articles were removed (Figure 1). Based on the inclusion and exclusion criteria, an abstract search of 799 articles was done, and a manuscript search of 75 articles was done. We identified five RCTs and one prospective comparative study (Table 1 and Table 2) [15-20]. No arthroscopic-assisted bone graft studies were included in this study.

**Table 1:** General characteristics of the studies included in this article VBG: vascularized bone graft; NVBG: non-vascularized bone graft; RCT: randomized controlled trial; 1,2-ICSRA: 1,2-intercompartmental supraretinacular artery

Author	Year	Study design	Country	Type of VBG	Type of NVBG
Braga-Silva et al. [15]	2008	RCT	Brazil	1,2-ICSRA	Iliac crest
Ribak et al. [16]	2010	RCT	Brazil	1,2-ICSRA	Dorsal radius
Raju and Kini [17]	2011	RCT	India	Volar radius	Iliac crest
Caporrino et al. [18]	2014	RCT	Brazil	1,2-ICSRA	Dorsal radius
Fan et al. [19]	2023	RCT	Canada	1,2-ICSRA	Iliac crest
Tabrizi et al. [20]	2022	Prospective comparative study	Iran	1,2-ICSRA	Iliac crest

**Table 2:** Reported outcomes in the included studies ROM: range of motion; DASH: Disabilities of the Arm, Shoulder and Hand; Y: yes; N; no

Author	Union rate	Time to union	Grip strength	ROM	Functional outcome	Mayo Wrist Score	DASH
Braga-Silva et al., 2008 [15]	Y	Y	Y	Y	N	N	N
Ribak et al., 2010 [16]	Y	Y	N	N	Y	N	N
Raju and Kini, 2011 [17]	Y	Y	N	N	N	N	N
Caporrino et al., 2014 [18]	Y	Y	Y	Y	N	N	N
Fan et al., 2023 [19]	Y	Y	Y	Y	N	N	Y
Tabrizi et al., 2022 [20]	Y	N	Y	Y	N	Y	Y

Study Characteristics

**Figure 2:**
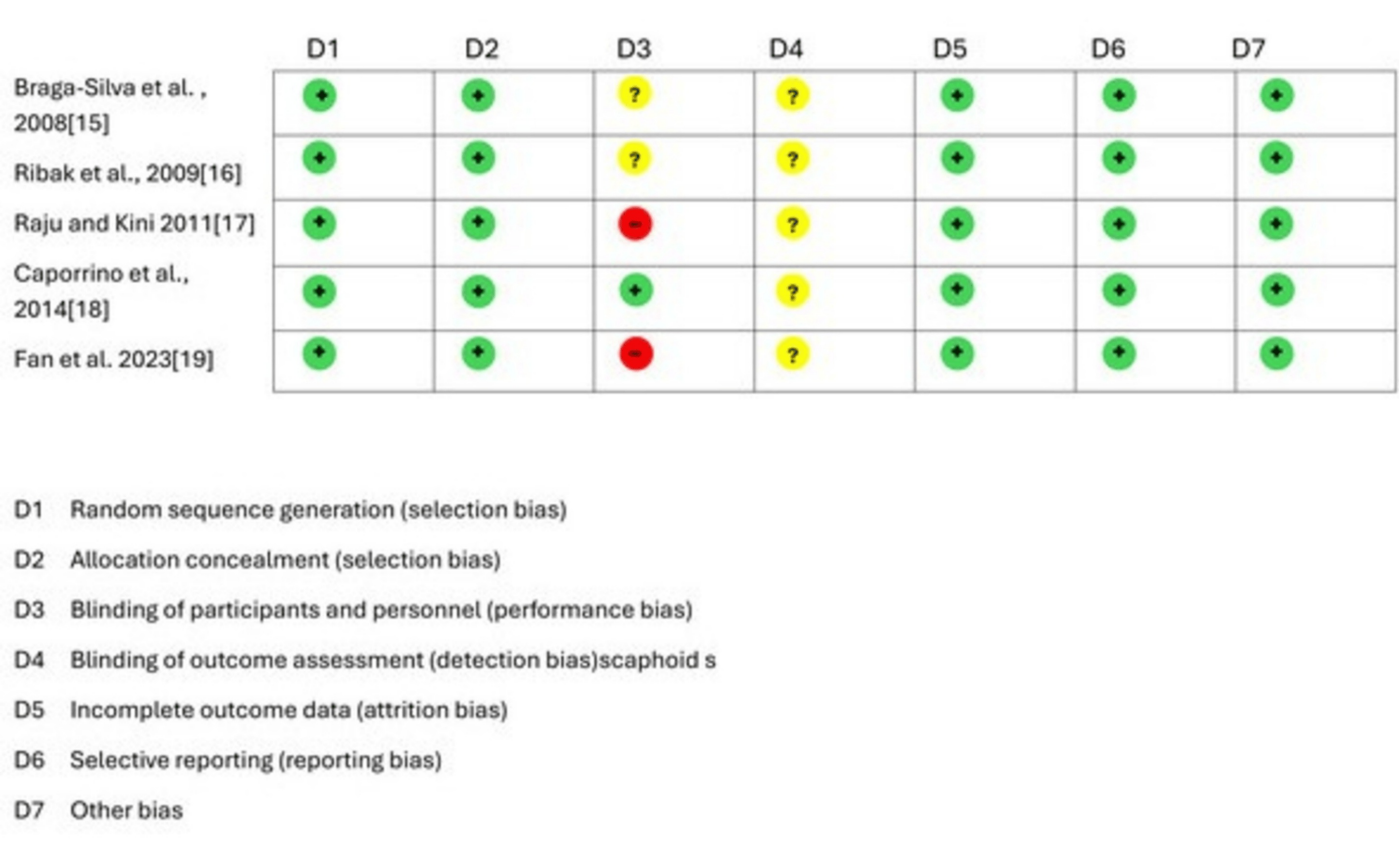
Risk of bias

Following a thorough evaluation, six independent studies, comprising five RCTs and one prospective comparative study, were included in the overall meta-analysis, with a total sample size of 337 patients. Among these, 167 patients received VBG, while 170 were treated with NVBG. Our study observed a male predominance, with the most common age group being individuals in their third decade of life, with the median third fracture being the most common site in both groups. There were no significant differences in the time interval from fracture to surgery between the two groups. Most studies in our meta-analysis had an average follow-up period of at least two years (Table 3 and Table 4). The methodological quality of RCTs is shown in Figure 2 to show the risk of bias. Most of the trials have a low risk of bias.

**Table 3:** Detailed characteristics of the patients in the articles included in the meta-analysis VBG: vascularized bone graft; NVBG: non-vascularized bone graft

Author	Total participant	Participant VBG	Participant NVBG	Age (mean)	Male	Female	Dominant hand VBG	Dominant hand NVBG	Time interval from fracture to surgery VBG (months)	Time interval from fracture to surgery NVBG (months)	Follow‑up time VBG (months)	Follow‑up time NVBG (months)
Braga-Silva et al., 2008 [15]	80	35	45	31	56	24	26	37	30	33.6	37.2	31.2
Ribak et al., 2010 [16]	86	46	40	NA	NA	NA	NA	NA	25.3	22.5	24.4	21.7
Raju and Kini, 2011 [17]	22	13	9	NA	NA	NA	NA	NA	NA	NA	NA	NA
Caporrino et al., 2014 [18]	73	35	38	27.7	71	2	NA	NA	36.9	27.6	29.4	28.6
Fan et al., 2023 [19]	48	25	23	22.47	43	5	8	6	17	18	NA	NA
Tabrizi et al., 2022 [20]	28	13	15	27.4	27	1	7	9	14.9	13.5	16	16.06

**Table 4:** Fracture site statistics of the included studies VBG: vascularized bone graft; NVBG: non-vascularized bone graft

Author	Median third (VBG)	Distal third (VBG)	Proximal pole (VBG)	Median third (NVBG)	Distal third (NVBG)	Proximal pole (NVBG)
Braga-Silva et al., 2008 [15]	23	12	NA	33	12	NA
Ribak et al., 2010 [16]	25	NA	21	22	2	16
Raju and Kini, 2011 [17]	4	3	6	3	2	4
Caporrino et al., 2014 [18]	23	NA	2	30	NA	8
Fan et al., 2023 [19]	NA	N A	7	NA	NA	2
Tabrizi et al., 2022 [20]	10	NA	3	9	NA	6

Union Rate

**Figure 3:**
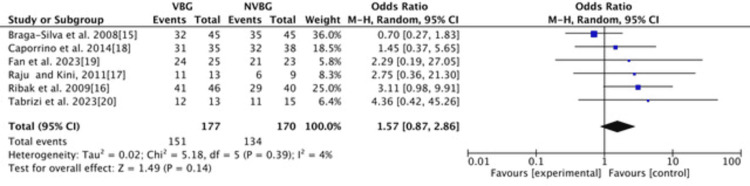
Forest plot of union rate VBG: vascularized bone graft; NVBG: non-vascularized bone graft

**Figure 4:**
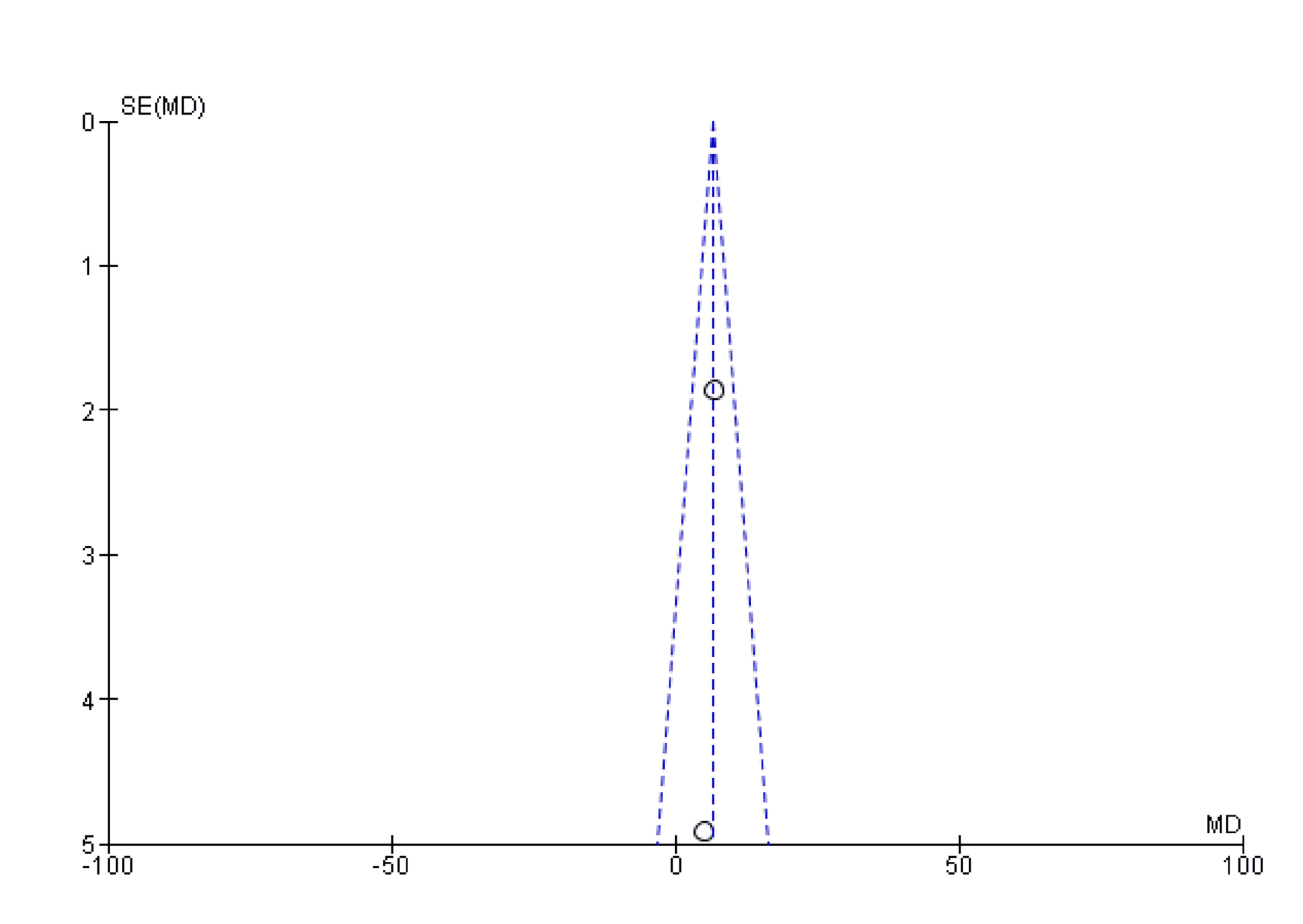
Funnel plot of union rate SE: standard error; MD: mean difference

The combined odds ratio is 1.57 (95% CI: 0.87-2.86), suggesting that the experimental group (VBG) may have a higher union rate than the control group (NVBG). However, since the confidence interval crosses 1 (0.87-2.86), this result is not statistically significant (Figure 3 and Figure 4).

Time to Union

**Figure 5:**
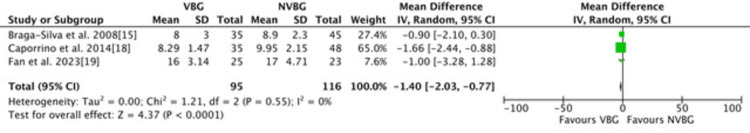
Forest plot of time to union VBG: vascularized bone graft; NVBG: non-vascularized bone graft

**Figure 6:**
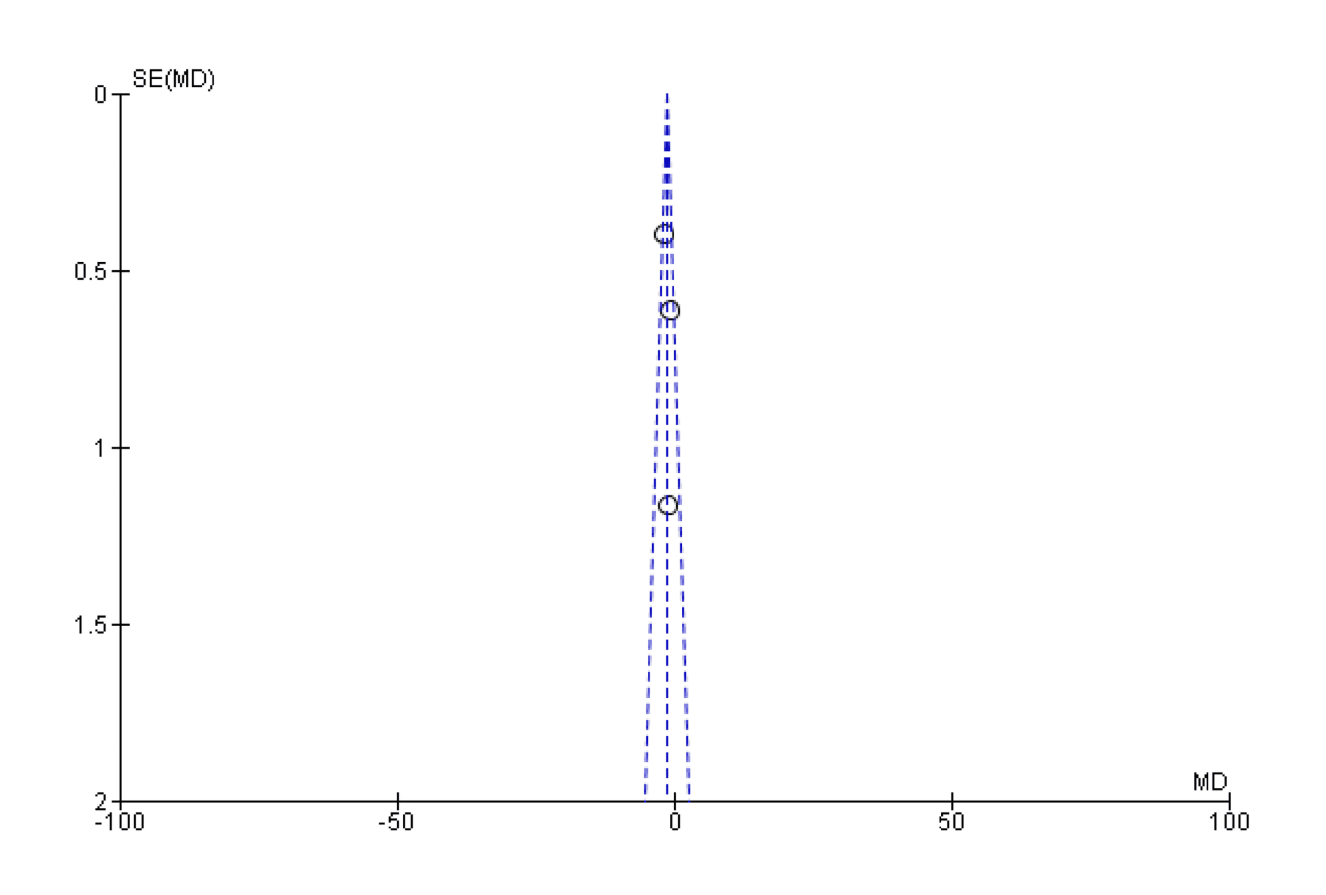
Funnel plot of time to union SE: standard error; MD: mean difference

The overall pooled mean difference is -1.40 (95% CI: -2.03 to -0.77), indicating that the experimental group (VBG) has a significantly shorter time to union compared to the control group (NVBG). The negative mean difference implies that VBG accelerates this outcome relative to NVBG (Figure 5 and Figure 6).

Radial Deviation

The forest plot presented compares radial deviation outcomes between the experimental (VBG) and control (NVBG) groups across three studies. The mean radial deviation in the experimental group varies between studies, with Braga-Silva et al. [15] reporting a mean of 6.8 degrees, Caporrino et al. [18] reporting 12.6 degrees, and Fan et al. [19] reporting 11 degrees. In contrast, the control group shows slightly different values, with means of 6.7 degrees for Braga-Silva et al. [15], 15.2 degrees for Caporrino et al. [18], and 10 degrees for Fan et al. [19]. The overall mean difference between the groups is -0.57 degrees (95% CI: -2.53, 1.38), indicating a slight non-significant trend towards better radial deviation in the control group. The heterogeneity (I2=55%) suggests moderate variability among the study results, but it is not statistically significant (p=0.11). The overall effect (Z=0.58; p=0.57) shows no significant difference between the experimental and control groups, suggesting that the treatment under investigation does not have a notable impact on radial deviation when compared to the control group.

**Figure 7:**
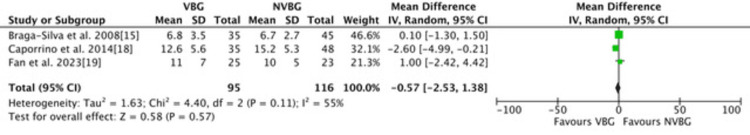
Forest plot of radial deviation VBG: vascularized bone graft; NVBG: non-vascularized bone graft

**Figure 8:**
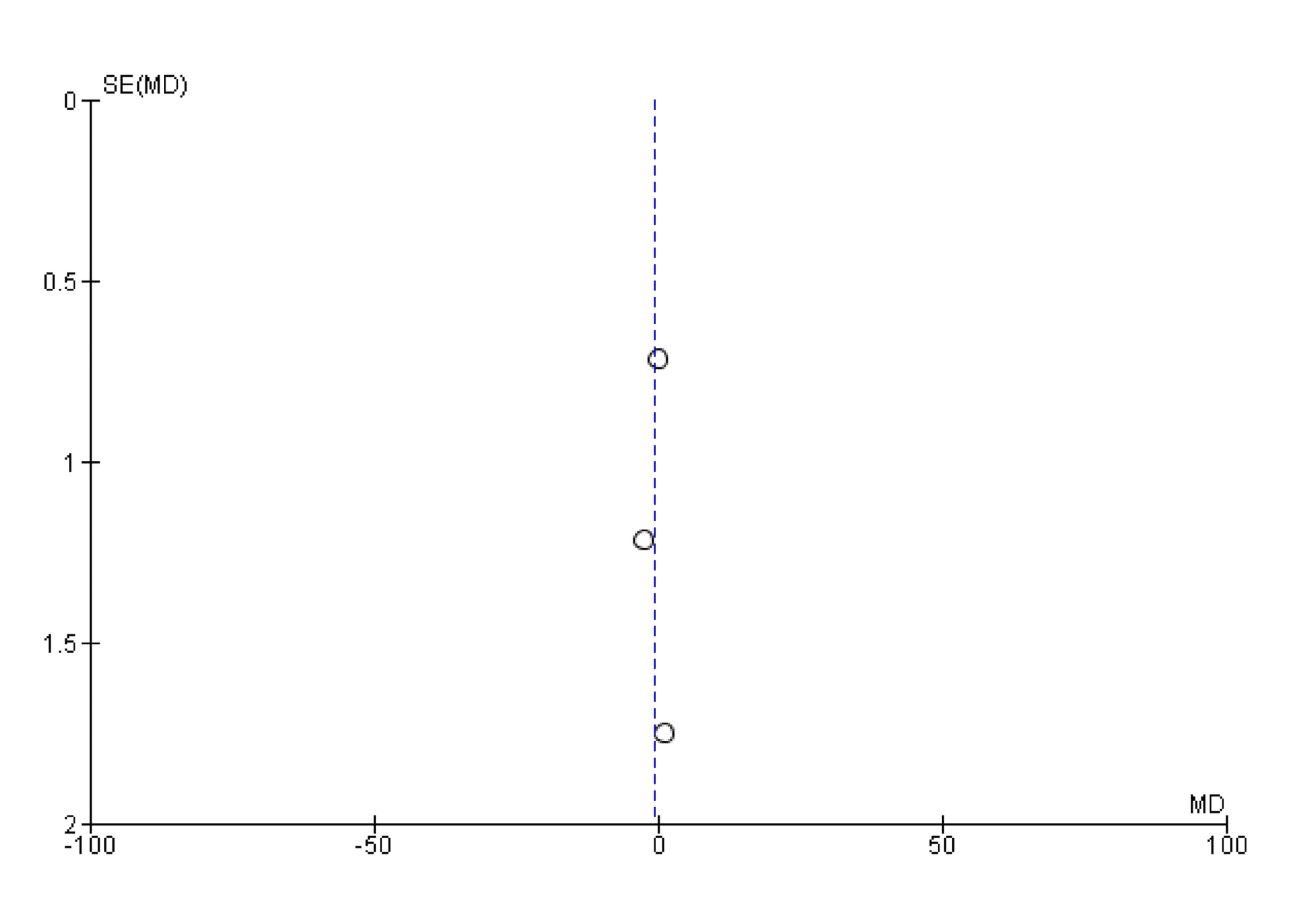
Funnel plot of radial deviation SE: standard error; MD: mean difference

Each study's result contributes differently to the pooled effect, with Caporrino et al. [18] showing a statistically significant benefit in radial deviation for the control group (-2.60; 95% CI: -4.99, -0.21), while the other two studies report no significant difference. Overall, this analysis suggests there is no clear advantage of the experimental treatment (VBG) in improving radial deviation compared to the control (NVBG) across these studies (Figure 7 and Figure 8).

Ulnar Deviation

The forest plot shown compares ulnar deviation between the VBG and NVBG groups across three studies. The mean ulnar deviation in the VBG group ranges from 23.4 to 25.4 degrees, whereas the NVBG group shows a range of 21.8-29.4 degrees.

The overall mean difference is -0.19 degrees (95% CI: -1.94, 1.55), indicating no significant difference between the two groups in terms of ulnar deviation. The heterogeneity (I2=72%; p=0.03) indicates substantial variability between the study results, which could be explained by different methodologies, patient populations, or interventions. The overall effect (Z=0.22; p=0.83) is not statistically significant, suggesting that there is no clear advantage of VBG over NVBG in improving ulnar deviation.

Looking at individual studies, Braga-Silva et al. [15] show a slight but non-significant benefit for the VBG group (1.60; 95% CI: -0.75, 3.95), while Caporrino et al. [18] demonstrate a statistically significant advantage for the NVBG group (-4.00; 95% CI: -7.37, -0.63). Fan et al. [19] report no difference between the groups (0.00; 95% CI: -4.08, 4.08).

**Figure 9:**
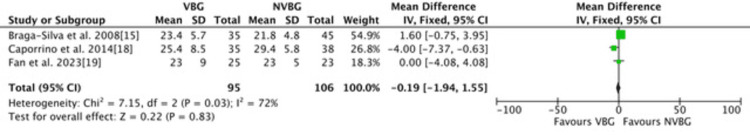
Forest plot of ulnar deviation VBG: vascularized bone graft; NVBG: non-vascularized bone graft

**Figure 10:**
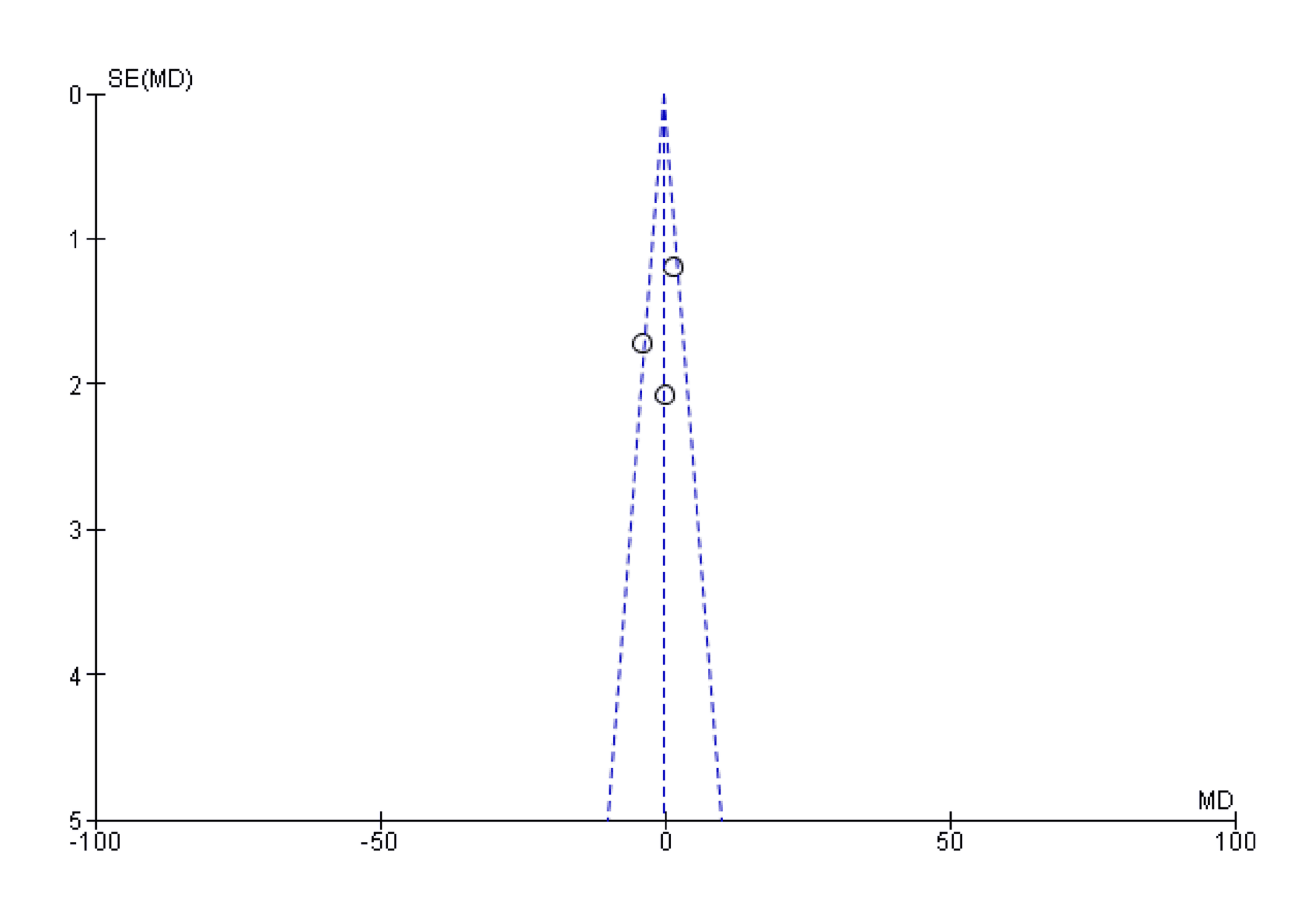
Funnel plot of ulnar deviation SE: standard error; MD: mean difference

In conclusion, while the analysis shows some variability in outcomes, there is no significant overall difference between the VBG and NVBG groups regarding ulnar deviation (Figure 9 and Figure 10).

Flexion

**Figure 11:**
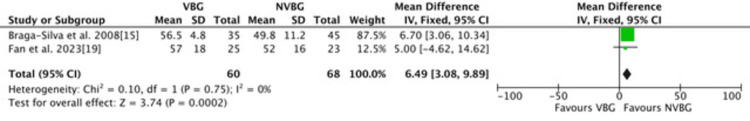
Forest plot of flexion VBG: vascularized bone graft; NVBG: non-vascularized bone graft

**Figure 12:**
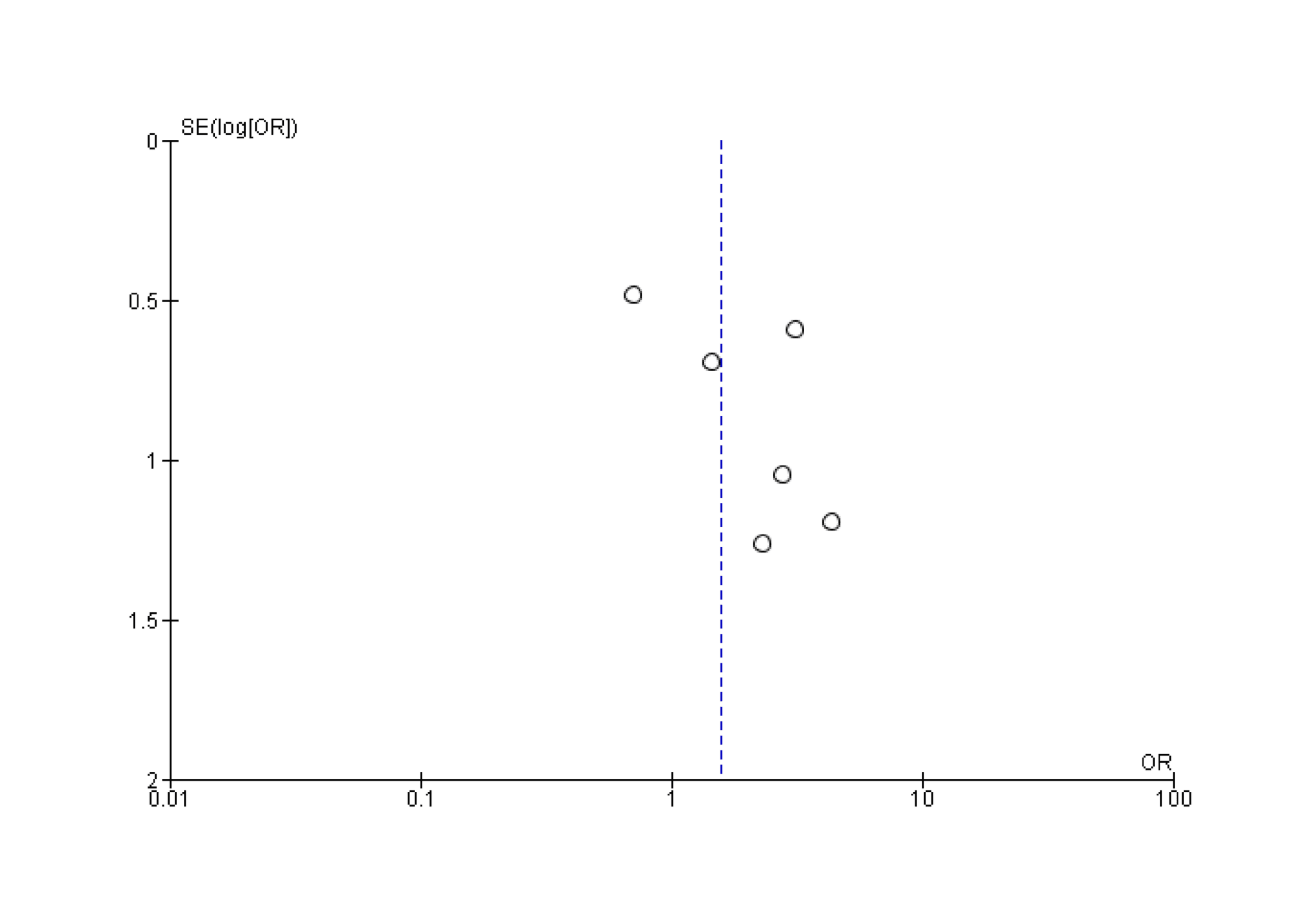
Funnel plot of flexion SE: standard error; MD: mean difference

The overall pooled mean difference is 6.49 (95% CI: 3.08-9.89), suggesting that the experimental group (VBG) achieves a statistically significant higher mean flexion compared to the control group (NVBG). This is supported by the fact that the confidence interval does not cross zero, indicating a significant effect (Figure 11 and Figure 12).

Extension

**Figure 13:**
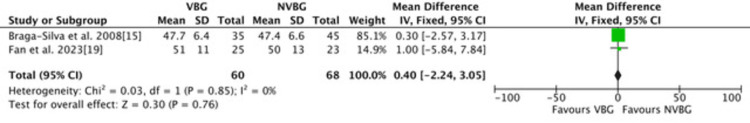
Forest plot of extension VBG: vascularized bone graft; NVBG: non-vascularized bone graft

**Figure 14:**
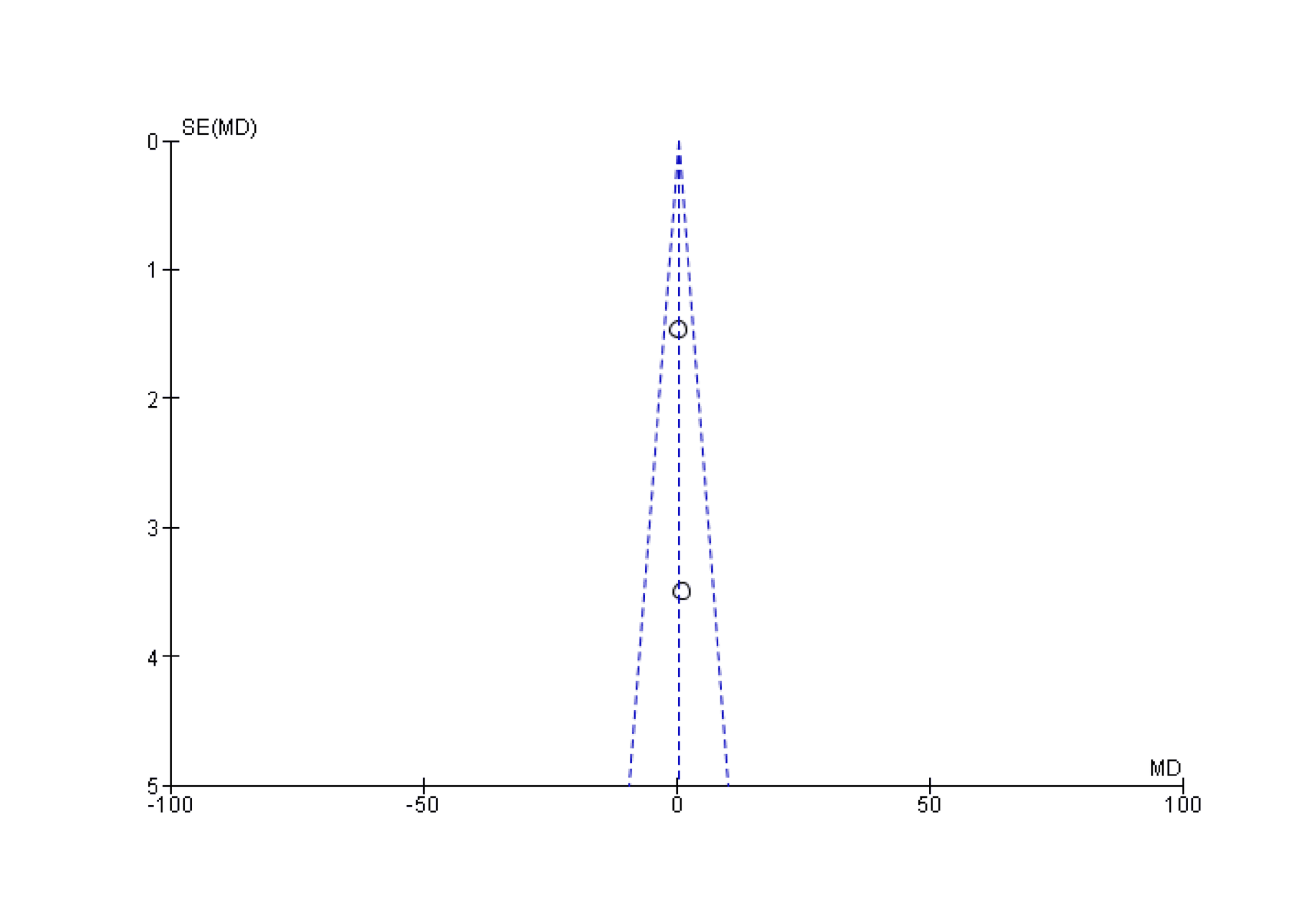
Funnel plot of extension SE: standard error; MD: mean difference

This meta-analysis shows no statistically significant difference in measurement between the VBG and NVBG groups. The overall mean difference is small (0.40), and the confidence interval for both individual studies and the combined result includes zero, implying that there is no clear advantage of one group over the other. The absence of heterogeneity (I2=0%) suggests that the results are consistent across the included studies (Figure 13 and Figure 14).

Discussion

The scaphoid is the most common carpal bone to be fractured. It is often associated with non-union. Open reduction and internal fixation with bone grafting is the recommended treatment for scaphoid non-union. Mathoulin and Haerle [21] reported that palmar carpal artery-based VBG showed earlier union with good functional outcomes. There are multiple graft approaches, including NVBG [22-24], dorsal and volar pedicled VBG [25-27], and free VBG [28-30]. VBG is widely used to treat scaphoid non-union. In theory, this technique re-establishes blood supply and promotes healing. Antoniou et al. evaluated the viability of the pedicle by MRI and reported revascularization in all 14 cases [31]. Pedicled VBG is technically more difficult and has a higher risk of failure, as obtaining an adequate size and shaped graft attached with a pedicle to fill a defect and correct a deformity is challenging [32].

In this meta-analysis, we compared radiological and functional outcomes following two techniques, VBG and NVBG, for scaphoid non-union. A total of six studies involving 377 patients with scaphoid non-union who underwent treatment with either VBG or NVBG were analyzed. Our results indicate a significant difference in time to union in favor of VBG. However, no significant functional advantage was shown. This is consistent with previous studies. Merrell et al. [10] determined that the union rate of VBG was 88% among 34 patients and the union rate in non-vascularized wedge graft was 47% among 30 patients for scaphoid non-union treatment. In a separate study, Ferguson et al. [33] analyzed 5,464 cases of scaphoid non-union outcomes from 144 studies. They reported union rates of 84% for VBGs and 80% for NVBGs. For patients with AVN, the union rates were 74% with VBGs and 62% with NVBGs. A multicentric retrospective study of 806 patients with scaphoid non-union by Ammori et al. [34] demonstrated that functional outcome is not influenced by graft type.

## Conclusions

This meta-analysis assessed the radiological and functional outcomes of VBG and NVBG for scaphoid non-union. VBG had a shorter time to union, suggesting superior biological potential due to enhanced vascularity. There was no significant difference in terms of functional outcomes between the two groups. Further, the treatment option should consider patient factors and their comorbidities, the location and duration of the non-union, and the surgeon's expertise. These findings suggest that while VBG offers certain advantages, its benefits should be interpreted with caution. Less invasive approaches, such as NVBG, may still yield effective outcomes and remain a valuable option for appropriate patients. Some limitations exist in the current meta-analysis. It does not report the functional outcomes, as each study used different tools for measurement. This study did not report outcomes on the basis of the type of fixation as adequate data were not available. The relation between non-union and fracture site could not be established.
